# Profiling of Insulin-Like Growth Factor Binding Proteins (IGFBPs) in Obesity and Their Association With Ox-LDL and Hs-CRP in Adolescents

**DOI:** 10.3389/fendo.2021.727004

**Published:** 2021-07-28

**Authors:** Abdur Rahman, Maha M. Hammad, Irina Al Khairi, Preethi Cherian, Reem Al-Sabah, Fahd Al-Mulla, Mohamed Abu-Farha, Jehad Abubaker

**Affiliations:** ^1^Department of Food Science and Nutrition, College of Life Sciences, Kuwait University, Kuwait City, Kuwait; ^2^Biochemistry and Molecular Biology Department, Dasman Diabetes Institute, Kuwait City, Kuwait; ^3^Department of Community Medicine and Behavioural Sciences, Faculty of Medicine, Kuwait University, Kuwait City, Kuwait; ^4^Genetics and Bioinformatics Department, Dasman Diabetes Institute, Kuwait City, Kuwait; ^5^Special Services Department, Dasman Diabetes Institute, Kuwait City, Kuwait

**Keywords:** adolescents, high sensitivity C-reactive protein, insulin-like growth factor binding proteins, obesity, oxidized low-density lipoprotein

## Abstract

Insulin-like growth factor binding proteins (IGFBPs) are critical modulators of metabolism. In adults, IGFBPs are associated with obesity and insulin resistance. However, the association of IGFBPs with metabolic homeostasis in children and adolescents is not yet fully characterized. In this study we investigated the association of plasma IGFBPs (IGFBP-1, 3 and 7) with weight, central adiposity and cardiovascular disease markers Hs-CRP and Ox-LDL. A total of 420 adolescents (age 11-14 years) were recruited from public middle schools in Kuwait. IGFBPs were measured using bead-based multiplexing while Hs-CRP and Ox-LDL were measured using ELISA. Results showed that levels of IGFBP-1 were significantly lower in obese and overweight children when compared to normal weight children. Correlation analysis showed negative association between the level of IGFBP-1 and waist circumference to height (WC/Ht) ratio. IGFBP-1 level was also negatively associated with Hs-CRP. It was also observed that the levels of IGFBP-3 and IGFBP-7 were negatively correlated with Ox-LDL. Our data demonstrate a strong negative association of IGFBP-1 with overweight/obesity, and the inflammatory marker Hs-CRP. This was not seen with the levels of IGFBP-3 and 7. The association of IGFBP-1 with central adiposity (WC/Ht ratio) was stronger than its association with BMI-for-age z-score. Therefore we suggest that IGFBP-1 could potentially be used as a sensitive biomarker for obesity and its subsequent effects in screening and monitoring of obesity-related metabolic complications in adolescents.

## Introduction

Childhood obesity is a public health concern. The Arabian Gulf region, including Kuwait, has one of the highest reported rates of childhood obesity (1). The prevalence of overweight and obesity among school children in Kuwait has been reported to be 45% (2, 3). Obesity has a complex etiology. The major contributing factors include both genetics and lifestyle behaviors such as poor dietary habits and lack of physical activity (4). Several reports show that childhood and adolescence obesity most likely persists into adulthood (5). Obesity is associated with many complications including dyslipidemia, hypertension, heart failure, and atherosclerotic cardiovascular diseases (6–8). Because of the increased health care cost to deal with these chronic diseases and the poor quality of life for patients, tackling obesity in early life should be a public health priority.

The need for a shift from reactive to predictive, preventive and personalized medicine (PPPM) is eminent (9, 10). While precision/personalized medicine has limited applications due to several challenges, tremendous effort is directed towards the advancement and use of this approach in the general clinical practice. One of the ways through which targeted therapy may be utilized is by screening for novel and easy-to-measure molecular biomarkers. The metabolic syndrome and its related pathologies, especially obesity, are in heightened need for such therapy options since they cause tremendous economic burdens on the healthcare systems (11). Identifying novel biomarkers, which could be used in detecting metabolic complications associated with obesity, is important for risk stratification and for monitoring and evaluating intervention programs. Personal variability related to genes, environment and lifestyle are taken into account when precision medicine approach is considered to be used (9).

Like insulin, insulin-like growth factors (IGFs) regulate diverse physiological functions related to growth, development and glucose homeostasis, which occur through common signaling pathways (12). When IGF-I and IGF-II were first described in the early fifties, they were presented as skeletal growth factors responsive to pituitary growth hormones (GH) and involved in the regulation of whole-body growth. Later investigations revealed that these molecules display homology to proinsulin and that they are regulated by multiple factors other than GH. The transport, metabolism and signaling by the IGFs are modulated by a family of binding proteins, which is comprised of six IGF binding proteins (IGFBP-1 – 6) as well as the IGFBP-related proteins (IGFBP-rP1, designated as IGFBP-7) (12–14). The different IGFBPs poses distinct features that allow them to specifically bind to certain receptors or translocate to various cellular compartments to mediate IGF-independent actions (14). Furthermore, different tissues produce different IGFBPs and their functions vary according to the metabolic conditions surrounding them (15). IGFBPs have been implicated in the development and pathogenesis of obesity and its related comorbidities like diabetes, metabolic syndrome and cardiovascular diseases (CVDs) through IGF-dependent as well as IGF-independent roles (12, 13).

Despite their significant sequence homology and their common ability to bind to IGFs with similar affinity, IGFBPs have unique structural features and distinct functions. This is mainly due to the different functional motifs of the family members. For examples, IGFBP-1 has an integrin-binding RGD (Arginine, Glycine, and Aspartate) motif and therefore can mediate cell migration. IGFBP-3 on the other hand does not have this RGD motif, but has many others including a nuclear localization sequence and a heparin binding domain (14).

The association of IGFBPs with obesity, metabolic syndrome and diabetes has been the subject of many investigations. Of these, IGFBP-1 has been consistently shown to be inversely associated with overweight and obesity (16–18), plasma insulin and glucose levels (19, 20), as well as fasting plasma leptin levels (21). Low serum IGFBP-1 levels have been reported to be predictive of the development of diabetes (22–24). The association of IGFBP-1 with metabolic homeostasis has been consistent across gender, different age groups and across various ethnicities (21, 25).

IGFBP-3 is the most abundant protein among all the IGFBPs and it transports more than 90% of IGF-I and IGF-II in circulation as a ternary complex with IGFs (12). However, its association with weight, metabolic syndrome and glucose homeostasis has not been consistent across studies. Some studies reported increased level of IGFBP-3 in overweight and obese subjects (16), while others reported no association with weight (26). In a prospective case-control study conducted on female nurses, a positive correlation of IGFBP-3 with the development of diabetes, BMI and waist circumference was reported (27).

IGFBP-7 is the most recent addition to the IGFBPs family. It has a similar amino acid sequence and structure to other human IGFBPs and can specifically bind to IGF-I and IGF-II. It is the least studied member of the IGFBP family in the context of its association with metabolic homeostasis. The available literature suggests that its serum levels are positively associated with BMI (28) and type 2 diabetes (29), fasting glucose levels (30), insulin resistance and metabolic syndrome (28, 31). A recent study reported higher levels of IGFBP-7 in coronary artery disease (CAD) patients compared to healthy subjects (32). In addition, IGFBP-3 was reported to be negatively associated with the levels of some inflammatory markers including C-reactive protein and interleukin-6 (33), whereas high levels of serum IGFBP-7 were associated with increased CRP levels (28).

The aim of this study was thus to investigate the association of IGFBP-1, 3 and 7 with weight status, waist circumference and well-established CVD risk factors, specifically Hs-CRP and Oxidized Low-Density Lipoprotein (Ox-LDL) in a group of healthy adolescents from Kuwait.

## Materials and Methods

### Study Participants

This is a cross-sectional study that was conducted in selected public middle schools from the State of Kuwait as previously described (34–36). Study participants were adolescents in the age range of 11–14 years. Data on socioeconomic status and other covariates were collected from the parents through a self-administered questionnaire, and from the adolescents using face-to-face interview.

### Ethics Statement

The study was approved by The Ethics Committee at Ministry of Health, Kuwait (No: 2015/248), the Ethics Committee of the Health Sciences Centre, Kuwait University (No: DR/EC/2338) and the Ethical Review Committee at Dasman Diabetes Institute (RA2017-026). Written informed consent was obtained from the parents and verbal assent was obtained from all the study subjects. We certify that the work conducted in this research complies with the ethical standards recommended by the Helsinki Declaration.

### Blood Collection and Biochemical Analyses

A sample of venous blood (5 mL) was collected from each child in EDTA-containing tubes. Plasma was separated and stored at −80 °C till analysis. IGFBPs levels were assessed using multiplexing immunobead array platform according to the manufacturer’s instructions (R & D Systems). Median fluorescence intensities were collected on a Bioplex-200 system and data were processed using the Bio-Plex Manager Software version 6 (Bio-Rad), with five-parametric curve fitting. Hs-CRP concentrations were determined using ELISA (Hycult Biotech, Cat. # HK369) following manufacturer’s instructions. Optimal dilution was found to be 1:1000. Ox-LDL concentrations were determined using ELISA (Immundiagnostik AG, Germany, Cat. # K 7810) following manufacturer’s instructions. Optimal dilution was found to be 1:10.

### Anthropometric Measurements and Other Covariates

Standing height and bodyweight of the study participants were measured in a standardized manner, using digital weight and height scale (Detecto, Webb City, MO, USA) with the participants standing erect without shoes and wearing light clothes. BMI-for-age z-scores were calculated using WHO growth charts. Obesity was defined as BMI-for-age ≥ +3 Standard Deviation (SD), while overweight was defined as BMI-for-age > +2 SD and < +3 SD. Waist circumference (WC) was measured in the horizontal plane at the superior border of the right iliac crest to the nearest 0.1 cm with a non-stretchable tape by a trained data collector. Measurements were taken at the end of normal expiration; three readings were taken, and the average of the three was recorded. Care was taken to ensure that the tape was horizontal to the floor and touched the skin without compressing it. The ratio of waist circumference (cm) to height in centimeters (WC/Ht ratio) was calculated and the obesogenic waist was defined as a WC/Ht ratio of > 0.5 (37).

### Statistical Analysis

Data were analyzed using the Statistical Package for the Social Sciences (SPSS) for Windows version 26 (IBM Corp., Armonk, N.Y., USA). Data were log-transformed and checked for normality using SPSS. Data for the IGFBP in different weight status groups and in male and female were presented as bar graphs showing mean with standard deviation (SD). Association between IGFBPs and weight status categories was assessed by both univariable and multivariable linear regression adjusting for age and sex. The association of IGFBPs with Hs-CRP and Ox-LDL (both log-transformed) was also assessed by linear regression analysis. Levels of IGFBPs were categorized into tertiles and the odds of overweight/obesity (combined) were determined in various tertiles of IGFBPs using binary logistic regression without and with adjusting for age and sex. Mean differences in the IGFBPs across weight status groups, age groups and gender were assessed by one-way ANOVA and t-test for independent samples. A p-value of < 0.05 was considered as statistically significant.

## Results

### Study Population Characteristics

**Figure 1:**
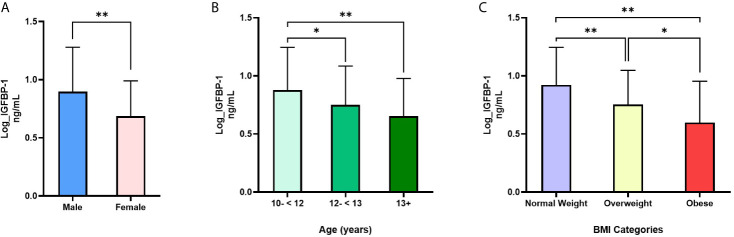
Differences in IGFBP-1 levels based on **(A)** sex, **(B)** age and **(C)** weight status: **(A)** IGFBP-1 are significantly lower in females compared to males; N=191 males, 228 females; t=6.18, p<0.001; **(B)** IGFBP-1 levels are significantly reduced with age; N=178 (10-<12), 143 (12-<13) and 98 (13+); **(C)** IGFBP-1 is reduced in overweight and obesity compared to normal weight; N= 198 normal weight, 88 overweight, 135 obese. Data is presented as means ± SD. Males and females were compared by t-test for independent samples, whereas, age groups and weight status groups were compared with one-way ANOVA. *p < 0.01; **p < 0.001.

**Table 1** summarizes the characteristics of the participants that were involved in the study. Data were analyzed for 420 participants of whom 192 (45.7%) were male. Mean (SD) age was 12.4 (1.5) years. Of the total samples, 47% adolescents were normal weight, 21% were overweight and 32% were obese. Median (IQR) for IGFBP-1 was 5.7 (3.5, 10.0) ng/mL, while median (IQR) for IGFBP-3 and IGFBP-7 were 835.0 (688.5, 1007.8) and 17.7 (15.2, 20.0) ng/mL, respectively. Female adolescents had significantly lower level of IGFBP-1 compared to males (p<0.001) (**Figure 1A**), whereas the differences in plasma levels of IGFBP-3 and IGFBP-7 between male and female subjects were non-significant (data not shown). IGFBP-1 level was significantly higher in the age group 10-<12 year old when compared to 12-<13 years (p<0.01) and 13+ years group (p<0.001) (**Figure 1B**). On the other hand, no significant difference was observed among different age groups in IGFBP-3 and IGFBP-7 levels (data not shown).

**Table 1:** Characteristics of the study population.

		N	Percentage
**Gender**	Male	192	45.7
	Female	228	54.3
**Nationality**	Kuwaiti	303	71.93
	Non-Kuwaiti	117	28.07
**Age groups**	10 - <12 years	179	42.5
	12 - <13 years	143	34.1
	13+ years	98	23.4
**Weight Status**	Normal weight	198	47.1
	Overweight	88	21.0
	Obese	134	31.9

### Association of IGFBPs and Weight Status

IGFBP-1 level was significantly lower in participants classified in the obese and overweight groups compared to normal weight children (p<0.01) (**Figure 1C**). No significant difference was observed in the levels of IGFBP-3 and IGFBP-7 when comparing different BMI categories (data not shown). Parallel to these results, the plasma level of IGFBP-1 was negatively associated with weight status (BMI categories) in univariable regression analysis [β (95% CI) = -0.16 (-0.20, -0.13)]. This association remained significant in the multivariable regression when adjusted for age and sex [β (95% CI) = -0.17 (-0.20, -0.13)]. The association of weight status with either IGFBP-3 or IGFBP-7 plasma levels was not significant either in multivariable regression, or in the adjusted multivariable regression (**Table 2**). In binary logistic regression, the odds of being overweight/obese (combined category) were significantly higher in the lower tertile of IGFBP-1 level when compared to the upper tertile (reference) [(OR (95% CI) = 6.82 (2.29, 20.30)] in the unadjusted model and this association sustained in the model adjusted for age and sex [(OR (95% CI) = 7.22 (3.92, 13.32)] (**Table 2**). IGFBP-3 and IGFBP-7 tertiles did not show significant association with overweight/obesity in binary logistic regression models, either unadjusted or adjusted.

**Table 2:** Association between IGFBPs and weight status in adolescents.

Linear Regression						
	β^1^	95% CI	p	β^2^	95% CI	p
IGFBP-1 (log)	-0.16	-0.20, -0.13	<0.001	-0.17	-0.20, -0.13	<0.001
IGFBP-3	-0.18	-35.40, 36.04	0.99	-0.18	-35.53, 35,18	0.78
IGFPB-7	0.02	-0.47, 0.51	0.93	0.02	-0.47, 0.50	0.78
**Logistic Regression**						
	**OR^1^**	**95% CI**	**p**	**OR^2^**	**95% CI**	**p**
**IGFBP-1**						
Lower Tertile	6.82	2.29, 20.30	<0.001	7.22	3.92, 13.32	<0.001
Middle Tertile	1.79	0.51, 6.26	0.25	1.53	0.83, 2.79	0.08
Upper Tertile	1.00	Ref		1.00	Ref	
**IGFBP-3**						
Lower Tertile	1.56	0.58, 4.18	0.38	1.57	0.58, 4.22	0.38
Middle Tertile	0.82	0.27, 2.51	0.50	0.83	0.27, 2.57	0.49
Upper Tertile	1.00	Ref		1.00	Ref	
**IGFBP-7**						
Lower Tertile	0.81	0.34, 1.94	0.37	0.80	0.33, 1.94	0.36
Middle Tertile	0.96	0.42, 2.22	0.67	0.98	0.42, 2.27	0.65
Upper Tertile	1.00	Ref		1.00	Ref	

^1^Unadjusted.

^2^Adjusted for age categories and sex.

In linear regression, IGF binding proteins (IGFBPs) were used as continuous variable (independent variable) and weight status (dependent variable) was categorized as normal weight, overweight or obese based on the WHO cutoffs of the BMI z-scores. N= 419 (IGFBP-1), 332 (IGFBP-3) and 428 (IGFBP-7). IGFBP-1 was log-transformed for normality.

Odds Ratios (OR) were calculated using binary logistic regression in which the response variable (weight status) was categorized into normal weight or overweight/obese, with the normal weight as the reference category.

### Association of IGFBPs With Waist Circumference

In the univariable regression analysis, the level of IGFBP-1 was negatively associated with WC/Ht ratio [β (95% CI) = -1.72 (-2.11, -1.33); p<0.001], and this association remained significant after adjusting for age and sex [β (95% CI) = -1.72 (-2.07, -1.37), p<0.001]. IGFBP-3 and IGFBP-7 levels did not show significant association with WC/Ht ratio either in the univariable or multivariable regression (**Table 3**). In the binary logistic regression, the odds of obesogenic waist, defined as a WC/Ht ratio of > 0.5, was significantly higher in the lower tertile of IGFBP-1 level when compared to the higher tertile [OR (95% CI) = 4.55 (2.74, 7.56)] in the unadjusted model. This remained significant in the model adjusted for age and sex [OR (95% CI) = 5.70 (3.26, 9.96)]. The tertiles of IGFBP-3 and IGFBP-7 levels did not show significant association with obesogenic waist in binary logistic regression models, whether they were adjusted or unadjusted (**Table 3**). When adjusted for age and sex, the negative association of IGFBP-1 level (log-transformed) with WC/Ht ratio [β (95% CI) = -1.73 (-2.08, -1.38)] was stronger than its association with the BMI-for-age z-scores [β (95% CI) = -0.11 (-0.13, -0.09)].

**Table 3:** Association of IGFBPs with waist-to-height ratio in adolescents.

Linear Regression						
	β^1^	95% CI	p	β^2^	95% CI	p
IGFBP-1 (log)	-1.72	-2.11, -1.33	<0.001	-1.72	-2.07, -1.37	<0.001
IGFBP-3	86.13	-301.12, 473.37	0.66	89.21	-299.35, 477.78	0.65
IGFPB-7	1.24	-4.03, 6.50	0.65	1.19	-4.08, 6.47	0.66
**Logistic Regression**						
	**OR^1^**	**95% CI**	**p**	**OR^2^**	**95% CI**	**p**
**IGFBP-1**						
Lower Tertile	4.55	2.74, 7.56	<0.001	5.70	3.26, 9.96	<0.001
Middle Tertile	1.26	0.78, 2.03	0.35	1.44	0.87, 2.39	0.16
Upper Tertile	1.00	Ref		1.00	Ref	
**IGFBP-3**						
Lower Tertile	1.05	0.62, 1.80	0.84	1.06	0.62, 1.80	0.84
Middle Tertile	1.06	0.62, 1.81	0.83	1.05	0.62, 1.80	0.85
Upper Tertile	1.00	Ref		1.00	Ref	
**IGFBP-7**						
Lower Tertile	0.77	0.48, 1.23	0.28	0.77	0.48, 1.24	0.28
Middle Tertile	1.10	0.69, 1.76	0.68	1.11	0.69, 1.77	0.68
Upper Tertile	1.00	Ref		1.00	Ref	

^1^Unadjusted.

^2^Adjusted for age categories and sex.

In linear regression, IGF binding protein (IGFBP) and WC/Ht ratio were used as continuous variables N= 419 (IGFBP-1), 332 (IGFBP-3) and 428 (IGFBP-7). IGFBP-1 was log-transformed for normality.

Odds Ratios (OR) were calculated using binary logistic regression in which the response variable (WC/Ht ratio) was categorized into non-obesogenic waist (WC/Ht ratio ≤ 0.5 and obesogenic waist (WC/Ht ratio > 0.5), with the normal non-obesogenic waist as the reference category.

### Association of IGFBPs With Markers of Oxidative Stress

**Figure 2:**
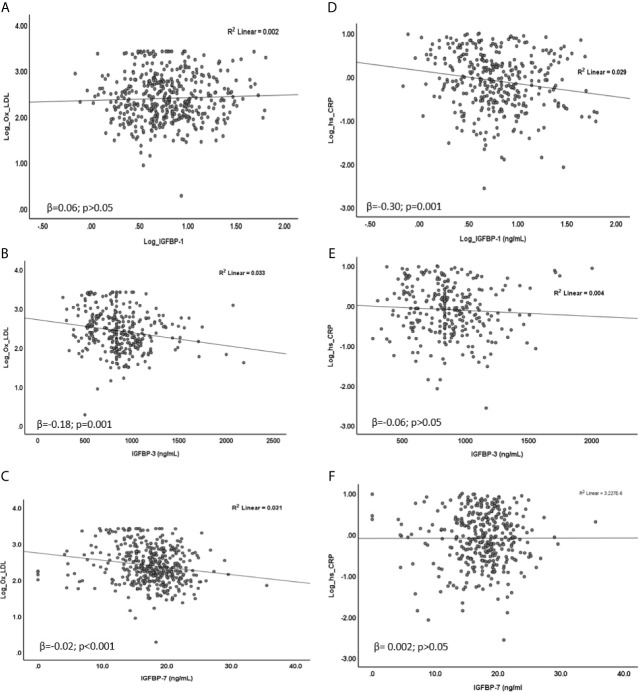
Association of Ox-LDL **(A–C)** and Hs-CRP **(D–F)** with IGFBPs. **(A)** Ox-LDL was not associated with IGFBP-1 (β = 0.06; p = 0.40), **(B, C)** Ox-LDL was negatively associated with IGFBP-3 (β = -0.18; p = 0.001) and IGFBP-7 (β = -0.02; p < 0.001). **(D)** Hs-CRP was negatively associated with IGFBP-1 (β = -0.30; p = 0.001) **(E, F)** No significant association of Hs-CRP was found with either IGFBP-3 (β = -0.06; p > 0.05) or IGFBP-7 (β = 0.002; p > 0.05). Data were analyzed by linear regression.

**Figure 3:**
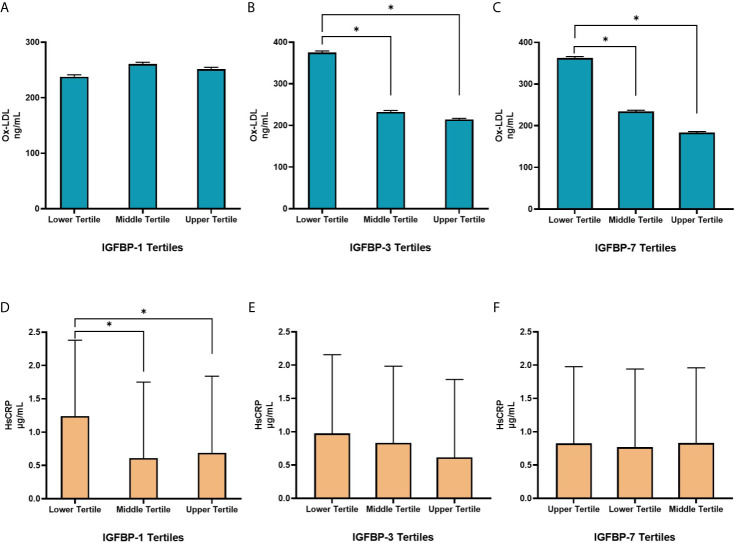
Distribution of Ox-LDL **(A–C)** and Hs-CRP **(D–F)** in different tertiles of IGFBPs. **(A)** Levels of Ox-LDL across the three tertiles of IGFBP-1 were not significantly different. **(B, C)** The levels of Ox-LDL were significantly higher in the lower tertiles of IGFBP-3 and IGFBP-7 compared to the middle and higher tertiles, no differences were observed between Ox-LDL levels in the middle and upper tertiles. **(D)** Levels of Hs-CRP were significantly higher in the lower tertile of IGFBP-1 compared to the middle and upper tertiles but were not different between the middle and upper tertiles. **(E, F)** Levels of Hs-CRP across the three tertiles of IGFBP-3 and IGFBP-7 were not significantly different. N=144 in each group. Data is presented as geometric means ± SD. *p < 0.01 by one way ANOVA with Bonferroni post-hoc comparison.

In linear regression analysis, plasma level of Ox-LDL was not associated with IGFBP-1 level (β=0.06; p=0.40). However, the level of Ox-LDL was negatively associated with the levels of IGFBP-3 (β=-0.18; p=0.001) and IGFBP-7 (β=-0.02; p<0.001) (**Figures 2A–C**). On the other hand, plasma level of Hs-CRP was negatively associated with the level of IGFBP-1 (β=-0.30; p=0.001). No significant association was found between the level of Hs-CRP with either the level of IGFBP-3 or the level of IGFBP-7 in plasma (**Figures 2D–F**). We further analyzed mean differences of Ox-LDL and Hs-CRP levels across different tertiles of the levels of IGFBPs. The results are shown in **Figure 3**. The level of Ox-LDL was significantly higher in the lower tertiles of IGFBP-3 and IGFBP-7 levels compared to the middle and higher tertiles, whereas no differences were observed between Ox-LDL level in the middle and upper tertiles (**Figures 3B, C**). The level of Ox-LDL across the three tertiles of the level of IGFBP-1 was not significantly different (**Figure 3A**). On the other hand, the level of Hs-CRP across the three tertiles of IGFBP-3 and IGFBP-7 levels was not significantly different (**Figures 3E, F**). However, the level of Hs-CRP was significantly higher in the lower tertile of IGFBP-1 level when compared to the middle and upper tertiles. Furthermore, the level of Hs-CRP was not significantly different between the middle and upper tertiles of IGFBP-1 level (**Figure 3D**).

## Discussion

In this study we investigated the association between obesity and the levels of various IGFBPs (particularly, IGFBP-1, 3 and 7) in a group of Kuwaiti adolescents. Our findings show that the plasma level of IGFBP-1 was decreased in children who were classified in the overweight or obese group. On the other hand, plasma levels of IGFBP-3 and IGFBP-7 were not affected. Furthermore, the level of IGFBP-1 was negatively associated with WC/Ht ratio. This association was stronger than the association of IGFBP-1 level with BMI-for-age z-scores. Interestingly, a strong negative association between the level of IGFBP-1 and Hs-CRP was also observed.

Childhood obesity is on the rising trajectory, especially in the Gulf region. Early predictors of obesity complications can contribute to the reduction in obesity-related metabolic consequences. Upper body obesity (truncal obesity) has been consistently shown to be more strongly associated with obesity-related comorbidities like diabetes and CVD. Thus, we investigated the association of truncal obesity, measured by WC/Ht ratio, with IGFBPs. Only IGFBP-1 was lower in the children with obesogenic WC/Ht ratio and the other two IGFBPs were not different in the two categories of WC/Ht ratio. To our knowledge, this is the first report to specifically correlate central obesity with IGFBP-1. The overall pattern of association was similar whether we categorized the study group based on weight using BMI or based on WC/Ht ratio. However, this correlation is stronger with WC/Ht ratio when compared to the BMI-for-age z-scores, which suggests that central body adiposity has a stronger influence on the level of IGFBP-1 than the overall body adiposity. Several other studies has supported this notion that central adiposity is more strongly associated with the adverse health consequences of obesity (38–41) and that WC/Ht ratio is a better indicator of adiposity than BMI (42–44).

Hs-CRP and Ox-LDL are well established cardiometabolic risk factors. In this study we found a differential association pattern between the level of the various IGFBPs under study with markers of oxidative stress (Ox-LDL) and cardiovascular diseases (Hs-CRP). For instance, the level of IGFBP-1 showed a negative association with Hs-CRP but not Ox-LDL. On the other hand, the levels of both IGFBP-3 and 7 were significantly associated with Ox-LDL but not Hs-CRP. Obesity is a state of low-level chronic inflammation. Thus, high Hs-CRP level in individuals living with overweight or obesity is consistent with this notion. The functional as well as the causal relationship between IGFBP-1 and Hs-CRP could not be deduced from this study. Therefore, a prospective study will possibly help delineate if the increased level of Hs-CRP are the cause or the consequence of lower level of IGFBP-1 in children with obesity.

A study conducted in Kuwait reported significantly lower levels of IGF-1 and IGFBP-3 in patients with coronary heart disease (CHD) and significant correlation between the level of IGFBP-3 and some metabolic markers including cholesterol, triglyceride (TG) and high-density lipoprotein (HDL) (45). Recently, a cross-sectional observational study conducted on 84 children under 10 years of age from two schools in Colombia demonstrated an inverse correlation of both IGFBP-1 and IGFBP-2 levels with the level of TG, as well as a direct correlation with HDL level (46). The study also reported lower level of IGFBP-1 with obesity. However, to the best of our knowledge, this is the first study to report an association of IGFBPs with Hs-CRP and Ox-LDL in adolescents. This is an important finding, since both markers are essential for detecting low inflammatory processes. It was interesting to observe that while the levels of both IGFBP-3 and -7 were not affected by obesity, they correlated negatively with Ox-LDL level. This suggests that the role of some IGFBPs in mediating inflammation and CVD can be independent of weight status and body fat composition. Further investigations are required to understand the role that both IGFBP-3 and -7 play in the development of CVDs. Our data further confirm that the different IGFBPs have distinct functional significance.

In this study the level of IGFBP-1 was negatively associated with age, which was not observed with the levels of both IGFBP-3 and 7. Although the age range of the study participants was very narrow (11-14 years), the difference in the level of IGFBP-1 was significant among various age groups. This suggests that IGFBP-1 is a sensitive biomarker when compared to the other IGFBPs. The age-dependent changes in IGFBP-1 level could be explained by changes in either body fat composition or in the level of sex hormones. The level of sex hormones is also affected by body fat composition. Therefore, body fat composition appears to be the major common determinant of IGFBP-1 level. The lower level of IGFBP-1 in girls compared to boys can also be explained by body fat composition, as girls generally have higher body fat percentage compared to boys at this age. However, the effect of changes in hormones should not be ruled out. Contrary to our results presented in this study, a Turkish group reported that serum level of IGFBP-3 are steadily increased in prepubertal children with age (47). However, due to lack of information regarding the pubertal stage of the subjects enrolled in our study, a direct comparison cannot be made. Nonetheless, such discrepancies emphasize the importance of personalized medicine and group-specific interventions. Since therapies that could work with children from Turkish descendent might not necessarily work on children from Kuwaiti (Arab) descendent. Therefore, further studies that cover a larger population with a wider age range are necessary to determine the exact interplay between growth, the level of sex hormones, body fat composition and the levels of various IGFBPs.

To our knowledge, this is the first study that investigated the association of IGFBPs with markers of inflammation and oxidative stress (Hs-CRP and Ox-LDL). Also, for the first time, we investigated the association of IGFBPs with a measure of central body adiposity. Most studies in this field use BMI as a measure of adiposity. Although BMI is generally a good indicator of overall body fat composition, it does not discriminate between upper and lower body fat composition. It is well documented that upper body fat composition (central adiposity) is more closely associated with obesity-related comorbidities. Therefore, the fact that IGFBP-1 was more strongly associated with WC/Ht ratio than the BMI z-scores makes IGFBP-1 a sensitive biomarker for obesity-related metabolic abnormalities. Other strengths of this study include a large sample size, which was representative of the adolescent population in Kuwait, using several statistical approaches to minimize the bias in the association, and the narrow age range of the study subjects. The latter is particularly important, as it will minimize the age-dependent influence on the association between obesity and IGFBPs. Furthermore, this study is based on healthy subjects without any overt obesity-related complications. Thus it highlights the possible use of IGFBP-1 as a screening marker for metabolic disorders. However, the limitations of this study include, the cross-sectional design, which does not allow us to establish causality, and the narrow age range of the participants, which could be considered as both a strength and a limitation. The limitation could result from the inability to project a similar association between IGFBP-1 and obesity into other age groups. Finally, due to logistic restrictions, a fasting blood sample could not be obtained, and thus data on parameters of glucose homeostasis and lipid profile are lacking.

In conclusion, the study results presented demonstrate the importance of IGFBPs in childhood obesity and highlight the distinct functions of the different members of the IGFBPs family. Since, a strong negative association was detected between the level of IGFBP-1 with overweight and obesity in adolescents. This negative association was shown to be more pronounced with central adiposity when compared to overall increased body weight. Furthermore, IGFBP-1 was negatively associated with Hs-CRP, which is a marker of inflammation. On the other hand, the levels of both IGFBP-3 and 7 were not associated with body weight. Nevertheless, the levels of these proteins showed a significantly negative association with the level of Ox-LDL, which is a marker of oxidative stress. Together these data further illustrate the possibility of using IGFBP-1 as a sensitive marker for obesity-related inflammation and its related comorbidities. The identification of such markers, especially in younger subjects, is a step towards the advancement in the field of Predictive, Preventive and Personalized Medicine.

## Data Availability Statement

The original contributions presented in the study are included in the article/supplementary material. Further inquiries can be directed to the corresponding authors.

## Ethics Statement

The studies involving human participants were reviewed and approved by The Ethics Committee at Ministry of Health, Kuwait (No: 2015/248), The Ethics Committee of the Health Sciences Centre, Kuwait University (No: DR/EC/2338), The Ethical Review Committee at Dasman Diabetes Institute (RA2017-026). Written informed consent to participate in this study was provided by the participants’ legal guardian/next of kin.

## Author Contributions

Conceptualization, AR, MA-F, and JA. Methodology, MH, IA, and PC. Software, AR. Validation, MH, IA, and PC. Formal analysis, AR. Investigation, AR, MA-F, and JA. Resources, AR, MA-F, and JA. Data curation, AR, MH, IA, and PC. Writing—original draft preparation, MH and AR. Writing—review and editing, AR, MH, RA-S., FA-M, MA-F, and JA. Supervision, MA-F and JA. Project administration, AR, MA-F, and JA. Funding acquisition, AR, MA-F, and JA. All authors contributed to the article and approved the submitted version.

## Funding

This work was supported by Dasman Diabetes Institute project No. RA 2017-026 and Kuwait University Project No. WF02/13.

## Conflict of Interest

The authors declare that the research was conducted in the absence of any commercial or financial relationships that could be construed as a potential conflict of interest.

## Publisher’s Note

All claims expressed in this article are solely those of the authors and do not necessarily represent those of their affiliated organizations, or those of the publisher, the editors and the reviewers. Any product that may be evaluated in this article, or claim that may be made by its manufacturer, is not guaranteed or endorsed by the publisher.
